# Auditory sustained potential as a biomarker of language functioning in children with autism and its implication in clinical trials

**DOI:** 10.3389/fpsyt.2026.1892698

**Published:** 2026-10-06

**Authors:** Vardan Arutiunian, Saeideh Davoudi, Ghizlane Gaougaou, Inga Sophia Knoth, Rae Buckser, Valérie Marcil, Sarah Lippé

**Affiliations:** 1 Azrieli Research Center of CHU Sainte-Justine, Montreal, QC, Canada; 2 Department of Neuroscience, University of Montreal, Montreal, QC, Canada; 3 Department of Nutrition, University of Montreal, Montreal, QC, Canada; 4 Department of Psychology, University of Montreal, Montreal, QC, Canada

**Keywords:** 40Hz auditory steady-state response, autism spectrum disorder, communication, language, sustained potential

## Abstract

**Objective:**

Most children with autism have language impairment, however, its neural mechanisms are not well understood. A potential biomarker associated with language in autism is a 40Hz Auditory Steady-State Response (ASSR) triggered by periodic click-trains which in electroencephalogram (EEG) evokes two types of responses – *40Hz steady-state gamma response* (ASSR) and *sustained potential* (SP). Although these responses represent low-level auditory processing, they correspond to different stages of sound perception/analysis and are essential in processing of spectrally/temporally complex sounds. However, until now there were no studies focusing on the potentials of these responses in clinical trials.

**Methods:**

The present open-label pilot study evaluated the effects of the probiotic beverage supplement Bio-K+ in children with autism (*N* = 19). Participants were assessed at three timepoints: T0 (baseline), T14 (14 weeks after intervention), and T22 (8 weeks after intervention, post-supplementation follow-up assessment), including EEG 40Hz ASSR and behavioral phenotyping.

**Results:**

First, at T0 we showed a reduction of SP amplitude in children with autism compared to controls, and this reduction was associated with lower speech/language/communication scores. Second, the amplitude of SP became significantly more negative during the probiotic supplementation period and, by T14, was no longer significantly different from the observed in TD controls. Finally, these changes in the amplitude of SP were associated with improvement in speech/language/communication scores.

**Conclusions:**

This study showed that the SP as well as its change was related specifically to speech/language/communication but not to other behavioral measures and has a potential to be used in future clinical trials.

## 1 Introduction

Autism Spectrum Disorder (ASD) is a neurodevelopmental condition affecting approximately 1 out of 31 8-year-old children according to the 2022 Autism and Developmental Disabilities Monitoring (ADDM) Network surveillance report (1) and being diagnosed based on behavioral symptoms in the domains of social communication and restricted and repetitive behaviors (2). Although language impairment is not a diagnostic criterion of ASD, most autistic children have language difficulties, varying in severity, i.e., from normal language skills (~25%) to severe language impairments with minimal or no verbal skills (3–6). However, neural mechanisms underlying these difficulties remain largely unexplored, and there is a need to identify reliable biomarkers of language impairment in ASD that can serve as objective measures in clinical trials.

Increasing evidence suggests that alterations of the microbiota–gut–brain axis may contribute to ASD symptomatology and co-occurring conditions through effects on neural development and brain function mediated by immune, metabolic, endocrine, and neural signaling pathways (7, 8). Consequently, *probiotic supplementation* has emerged as a potential therapeutic approach for ASD, with several studies reporting improvements in gastrointestinal symptoms as well as behavioral and communication outcomes, although the evidence remains mixed (9–11). Given the proposed influence of the microbiota–gut–brain axis on neural function, probiotic interventions may also affect neural and sensory processing, highlighting the need for objective neurophysiological biomarkers to evaluate intervention-associated changes.

It has been demonstrated that altered low-level sensory processing in the auditory cortex is associated with alterations in high-level communication skills in children with ASD, including language (12–17). One of the reliable paradigms to register this low-level sensory processing is 40Hz Auditory Steady-State Response (40Hz ASSR), where participants are presented with a click train or amplitude-modulated tones at a gamma frequency range (30–80Hz in magneto- or electroencephalography, MEG or EEG), usually at ~ 40Hz (18–20). Previous studies have shown that such stimuli activate neuronal populations in the primary and secondary auditory cortices and elicit two distinct EEG responses: (1) an oscillatory response at the stimulation frequency (typically 40 Hz), known as the ASSR, which is commonly quantified using inter-trial phase coherence (ITPC); and (2) a sustained potential (SP), characterized by a stable negative deflection in the evoked potential during sound presentation (18, 21–24). The advantage of the ASSR paradigm is that it is a passive methodology requiring no response from participants and has a high test-retest reliability (25, 26). This makes the paradigm well-suited to clinical trials, especially those which include non-verbal or minimally verbal individuals.

There are few studies on individuals with ASD using 40Hz ASSR, and the results are inconsistent. Some studies have reported a reduction of 40Hz auditory gamma response in children and adolescents with ASD, as well as in first-degree relatives of individuals with ASD (27–30) compared to typically developing (TD) controls. However, other studies have demonstrated no difference between children with and without ASD in this response (31–34). Some findings have also indicated that alterations in 40Hz ASSR can be related to language impairment in children with ASD (27, 35). These inconsistencies in results can be explained by the highly heterogeneous nature of the ASD population and the variability in language skills in ASD. That is, it has been shown that the key part of the neural circuitry responsible for entrainment to exogenous auditory stimuli involves parvalbumin positive (PV+) basket cells and the pyramidal neurons of the upper layers of the auditory cortex, and the occupation of a specific channel on those PV+ cells reduced 40 Hz auditory gamma response highlighting the specific molecular mechanism contributing to the strength of 40Hz ASSR (36). Therefore, some authors suggest that the reduction of 40Hz ASSR may be associated not with the whole ASD population, but rather with a specific genetic subgroup of individuals with ASD (36, 37).

Although SP is an auditory response evoked by the same periodic stimuli as the oscillatory steady-state response, it has not been widely investigated until recently. There is a very limited number of studies that have focused on this response in neurodevelopmental populations (ASD and Rett Syndrome), and they have consistently showed a reduction of the amplitude of SP (31, 34, 38). The authors proposed that SP is related to pitch processing as it can be triggered by any periodic spectrally complex sounds, including speech sounds (34, 38, 39), and, thus, the adequate functioning of SP is essential for speech perception and language processing. Therefore, a reduced SP could be a promising marker of speech and language impairments.

An important insight into the distinct cellular and system mechanisms of ASSR and SP comes from studies in animals and human neuroimaging research. Both neuronal findings in monkeys (40) and MEG studies in children and adults (23, 34) have shown similar results, such that the anatomical sources of ASSR and SP are distinct: while both auditory responses are generated by Heschl’s (transverse temporal) gyrus, oscillatory ASSR at a specific stimulation frequency is generated by the primary auditory cortex (A1) whereas the localization of SP is in the anterolateral region of Heschl’s gyrus (39), which represents a so-called ‘pitch processing center’ (41, 42). Evidence from electrophysiological recordings in animals suggested two distinct types of neurons in those regions with a wide representation of non-synchronized cell populations in the ‘pitch processing center’ that generates SP (40). This highlighted the differences in functional characteristics of ASSR and SP as these responses are generated not only by different areas of the auditory cortex, but also by different cell types. Given these differences, ASSR and SP reflected different stages of sound processing, where ASSR is a primary neural response at a specific frequency of stimulation and SP is a more complex response that integrates pitch information across different frequency units (40). Such integrative processing is particularly relevant for complex natural sounds, including speech, which require the integration of information across multiple frequency channels. Consequently, alterations in SP may have greater relevance to speech and language processing than alterations in the frequency-specific ASSR. It is important to note, however, that despite morphological and functional differences these neural responses reflect low-level auditory processing (43).

Although SP may have relevance for speech perception and language processing (31, 34, 38) and can be acquired from non-verbal/minimally verbal children, it has never been investigated in clinical trials. The aim of this exploratory open-label pilot study is to investigate, to our knowledge for the first time, the potential of SP as a neural biomarker by evaluating its group discriminative ability, its relation to speech/language/communication outcomes and its changes during intervention in association with the changes in caregiver-reported speech/language/communication scores. If SP is a sensitive biomarker of speech/language/communication variation in ASD, it should also be sensitive to individual variations resulting from intervention.

The present study is a 30-week open-label pilot study aiming to test the acceptability and the safety of the 3-strains probiotic beverage supplement (Bio-K+) and the feasibility of the proposed protocol in children with ASD (see (44) for the full protocol and study design). It includes multiple EEG-based measures collected at three timepoints (T0 = baseline, T14 = 14 weeks after the beginning of probiotic supplementation, T22 = 8 weeks after the end of the intervention, post-supplementation follow-up assessment), including ASSR paradigm. In the framework of the present study, we had several goals:

- To provide between-group comparisons (ASD vs. TD) in both 40Hz ASSR (measured with ITPC) and SP at the baseline timepoint (T0). The baseline cross-sectional analyses were designed to establish the relative clinical relevance of the conventional 40 Hz ASSR and SP within the same experimental paradigm before evaluating their longitudinal sensitivity to intervention-associated changes.
- To explore relationships between auditory responses (40Hz ASSR and SP) and behavioral assessments of speech/language/communication skills as well as other cognitive, sensory, and health measures in the ASD group at the baseline timepoint (T0).
- Effect of probiotic supplementation: to investigate the changes of 40Hz ASSR and SP after 14 weeks from the start of Bio-K+ probiotic product administration (T14) and at the post-intervention timepoint (T22) in comparison to baseline (T0) in the ASD group. Also, to reveal whether the changes in the auditory responses (40Hz ASSR and SP) were related to changes in behavioral measures in the ASD group during the probiotic supplementation period (from T0 to T14).

## 2 Materials and methods

### 2.1 Participants

A total of 128 EEG files (64 EEGs for 40Hz ASSR and 64 EEGs for SP) for three timepoints were analyzed, resulting in 19 children with ASD (5 female, age range 4–11 years, *M* = 7.10) at three timepoints and 10 TD controls (5 female, age range 4–11 years, *M* = 7.8) at one timepoint. There were no between-group differences in age, *t*(17.9) = –0.78, *p* = 0.44, and sex, χ^2^(1) = 0.75, *p* = 0.39.

Participants were recruited via advertisements on social media or medical charts. All children in the ASD group had a medical diagnosis of ASD established by experienced clinicians according to DSM-5 criteria, and the diagnosis was confirmed based on Autism Diagnosis Observation Schedule – Second Edition (45). Exclusion criteria were (1) autism in the context of genetic syndrome such as Fragile X or tuberous sclerosis complex, (2) cancer, diabetes or genetic disorder such as Down Syndrome 21 or 14; (3) immune system disorder; (4) intolerance or allergy to the probiotic beverage; (5) having taken probiotics during the previous three months; and (6) having taken antibiotics in the previous month. TD children had no history of psychiatric and/or neurodevelopmental disorders.

This study was approved by the ethics review board of the Centre Hospitalier Universitaire Sainte-Justine (#2021–3412). Informed consent was obtained from all parents involved in the study and participants gave their assent when possible.

The EEG analyses reported here were conducted as part of a 30-week open-label pilot intervention study evaluating the acceptability, safety, and feasibility of probiotic supplementation in children with ASD.

### 2.2 Bio-K+ probiotic supplement administration

Bio-K+ is a brand of commercially available and well-defined specific probiotic food that consists of a vegan pea-based, raspberry-flavored fermented drinkable product containing a minimum of 50 × 109 colony forming units (CFU) of three strains: *L. acidophilus* CL1285, *L. casei* LBC80R and *L. rhamnosus* CLR2. The product was manufactured and supplied by Kerry Canada inc. (Laval, Quebec, Canada). For the duration of the supplementation period, children with ASD consumed one bottle per day (98 g) all at once or over the course of the day. Children were allowed to mix the content of the probiotic beverage bottle with any cold beverage. Detailed acceptability, safety, and compliance data have been reported previously (44). Briefly, all participants accepted the probiotic beverage, no product-related adverse events requiring discontinuation were reported, and compliance was 100%, defined as consumption of the probiotic on at least 6 of 7 days per week throughout the 14-week supplementation period. All participants completed the supplementation protocol according to the study procedures.

### 2.3 Behavioral assessment

Each participant’s behavioral phenotype was assessed using standardized parent-completed questionnaires. We utilized the Autism Treatment Evaluation Checklist (46) completed by parents and used scores in the three domains: a) speech/language/communication; b) sociability; c) sensory/cognitive awareness, where higher scores indicate greater symptom severity. This checklist has been specifically developed to assess change in existing symptoms by comparing scores over time, making it suitable for clinical trial studies. Gastrointestinal (GI) symptoms were screened with the Gastrointestinal Severity Index (47), a questionnaire that measures nine components of GI distress, where higher scores indicate greater symptom severity. Sleep issues were assessed via the Children’s Sleep Habit Questionnaire (48), where a total score of 41 or more indicates that symptoms are clinically significant. Behavioral data were collected at all three study timepoints for the ASD group, whereas TD children completed the same assessments only once at baseline.

### 2.4 Experimental paradigm, EEG data collection and analysis

In total, each participant was presented with 50 auditory stimuli that evoked 40Hz ASSR in a random order with the other stimuli (n = 50, at another frequency, 6Hz). Participants were exposed to click trains (1.5 ms bursts of white noise, see (49)) presented at a frequency of 40 per second (40Hz) with a random inter-stimulus interval of 1–1.5 seconds. Auditory stimuli were presented through dual speakers positioned one meter from the participant on either side. As the auditory processing task did not require active attention, participants watched a movie with the sound muted and without subtitles during the recording to maintain wakefulness and minimize head movements.

At all timepoints, EEG was acquired with a high-density 128-channel net from Electrical Geodesics, Inc. (Magstim EGI, Eugene, OR, USA) in a darkened, soundproof room. The appropriate HydroCel Geodesic Sensor Net size was selected according to each child’s head circumference. Sponge-based electrodes were soaked in electrolyte solution prior to recording, eliminating the need for conductive gel. Signal was acquired using an EGI Net Amp 300 amplifier and saved on a G4 Macintosh desktop computer using NetStation EEG Version 4.5.4. Data were collected at a 1000Hz sampling rate, the vertex (Cz) was used as an online reference electrode, and impedances were maintained at 40 kΩ or below. To provide standardized EEG data processing, we used the Harvard Automated Preprocessing Pipeline for EEG (HAPPE4.0) for artifact detection, cleaning and rejection (50) developed specifically for pediatric populations with neurodevelopmental disorders and high-artefact data. After artefact removal and correction, 6 out of 128 EEG recordings were excluded from the analysis due to severely impacted signals. Consequently, the final analytical sample varied across analyses depending on EEG data quality, the availability of recordings at each timepoint, and the behavioral measures included in a given statistical model. The corresponding sample size is reported for each analysis in **Table 1**, which also presents the EEG quality metrics for participants who were included in the analysis (with valid EEG data after pre-processing).

**Table 1:** EEG quality metrics for participants who were included in the analysis (with valid EEG data after pre-processing).

Characteristics, M(SD)	40Hz auditory steady-state response				Sustained potential			
	TD	ASD			TD	ASD		
	N = 10	T0 (N = 19)	T14 (N = 18)	T22 (N = 15)	N = 10	T0 (N = 19)	T14 (N = 16)	T22 (N = 15)
Percent of good channels	93.4(3.8)	93.6(3.4)	94.3(3.1)	95.4(3.8)	78.6(11.7)	72.4(10.8)	74.7(9.2)	74.7(12.7)
Percent variance kept of post waveleted data	36.1(21.9)	10.9(13.1)	14.2(19.5)	22.7(25.0)	15.5(14.1)	7.5(8.0)	6.8(6.3)	9.2(11.0)
Percent of segments retained after processing	100.0(0.0)	99.7(1.0)	95.4(11.5)	99.7(0.5)	99.6(0.8)	83.3(21.2)	85.6(22.7)	86.7(21.4)
Percent ICs rejected	0.9(0.9)	0.8(1.2)	0.7(1.1)	0.8(1.3)	NA	NA	NA	NA

Offline band-pass filters of 1–100Hz for time-frequency analysis (to calculate 40Hz ITPC) and 0.1–9Hz for evoked potential analysis (to calculate SP) were used. The cleaned EEG data were cut in 3000ms epochs (ranging from –1500 to 1500ms) for 40Hz ITPC calculation and in 1600ms epochs (ranging from –300 to 1300ms) for SP calculation; baseline correction from –299 to 0ms was applied for both types of the analyses. To estimate 40Hz ITPC, we averaged ITPC-values in 0–1000ms time interval (stimulus presentation time) in 39–41Hz frequency range; ITPC can be values from 0.0 to 1.0, where higher values indicate higher consistency of phases across the trials (18). It has been shown that ITPC is a more reliable measure compared to the total power in the same frequency range (27). To estimate SP, we averaged the amplitude of this evoked potential in 200–600ms after stimulus onset. This time window was chosen based on the previous studies showing that the sustained part of the evoked response starts at around 200ms after stimulus onset and the initial part of this response (but not the later) is altered in ASD (31, 34). Both 40Hz ITPC and SP were calculated for the Fz electrode (#11 in Magstim EGI system) which captures auditory potentials on the scalp EEG (51).

### 2.5 Statistical analysis

Statistical analysis was done in R 2023.03.1 + 446 (52), using *lme4 v1.1.35.5* package (53); correction for multiple comparisons (false discovery rate, FDR) was applied to each set of the analyses, and *p*-values were corrected with the *p.adjust.method* in R. The data were plotted with *ggplot2 v3.5.2* (54), and the tables for model outcomes (**Tables 2**–**4**) were created with the *sjPlot v2.8.17* package (55). Figures representing neural responses were created with the python data visualization library *matplotlib v3.1.1* (56) for time-frequency maps and with the EEGLAB v2024.0 (57) for evoked potentials. The structure of the models will be specified further in the Results section.

**Table 2:** Changes in the scores of the main behavioral measures during treatment (T0 = baseline/treatment’s start, T14 = 14 weeks after T0/treatment’s end, T22 = 8 weeks after T14, post-treatment time).

Predictors	Speech/Language/Communication				Sociability				Sensory/Cognitive awareness				Gastrointestinal symptoms				Sleep, CSHQ			
	Estimate	SE	t	p	Estimate	SE	t	p	Estimate	SE	t	p	Estimate	SE	t	p	Estimate	SE	t	p
(Intercept)	9.96	1.95	5.10	<0.001***	11.13	1.45	7.66	<0.001***	11.68	1.46	8.02	<0.001***	4.61	0.55	8.42	<0.001***	49.30	2.05	24.04	<0.001***
Timepoint, T14	-2.02	0.80	-2.51	0.019*	-3.83	1.01	-3.78	0.001**	-4.17	1.10	-3.80	0.001**	-3.16	0.56	-5.59	<0.001***	-5.04	1.51	-3.33	0.003**
Timepoint, T22	-0.87	0.86	-1.02	0.316	0.54	1.08	0.50	0.620	-0.97	1.17	-0.83	0.411	-0.26	0.59	-0.44	0.660	-1.95	1.56	-1.25	0.220
Random effects																				
σ^2^	4.47				7.23				8.55				2.48				16.11			
τ_00 ID_	69.83				31.90				30.22				3.20				56.67			
ICC	0.94				0.82				0.78				0.56				0.78			
N _ID_	20				20				20				21				19			
Observations	45				45				45				48				45			
Marginal R^2^/Conditional R^2^	0.010/0.940				0.091/0.832				0.081/0.797				0.271/0.681				0.059/0.792			

Significance is labeled with *p < 0.05, **p < 0.01, ***p < 0.001 (significant *p*-values are FDR-corrected) and highlighted in bold.

**Table 3:** Associations between 40Hz Inter-Trial Phase Coherence (ITPC) and behavioral measures in children with Autism Spectrum Disorder (ASD) at T0.

Predictors	40Hz inter-trial phase coherence				40Hz inter-trial phase coherence, T0-T14 change value				
	Estimate	SE	t	p	Predictors, T0-T14 change score	Estimate	SE	t	p
(Intercept)	0.11	0.24	0.48	0.645	(Intercept)	-0.01	0.06	-0.23	0.823
Language	-0.02	0.01	-1.85	0.097	Language	0.02	0.02	1.04	0.327
Sociability	0.01	0.01	0.90	0.392	Sociability	0.00	0.01	0.44	0.673
Sensory/Cognitive	-0.00	0.01	-0.30	0.770	Sensory/Cognitive	-0.01	0.01	-0.92	0.383
Gastrointestinal symptoms	0.00	0.02	0.09	0.930	Gastrointestinal symptoms	-0.00	0.01	-0.41	0.696
Sleep	0.01	0.01	1.09	0.305	Sleep	-0.00	0.01	-0.32	0.757
Observations	15				Observations	14			
R^2^/R^2^ adjusted	0.451/0.146				R^2^/R^2^ adjusted	0.149/-0.382			

The relationship between T0–T14 change in 40Hz ITPC and T0–T14 change scores of behavioral measures in children with ASD.

**Table 4:** Associations between the amplitude of sustained potential (SP) and behavioral measures in children with Autism Spectrum Disorder (ASD) at T0.

Predictors	Sustained potential				Sustained potential, T0-T14 amplitude change				
	Estimate	SE	t	p	Predictors, T0-T14 change score	Estimate	SE	t	p
(Intercept)	-5.69	6.44	-0.88	0.400	(Intercept)	3.55	3.05	1.16	0.283
Language	0.72	0.23	3.10	0.025*	Language	3.39	1.08	3.13	0.033*
Sociability	0.25	0.21	1.19	0.266	Sociability	-0.19	0.38	-0.51	0.623
Sensory/Cognitive	-0.41	0.28	-1.47	0.176	Sensory/Cognitive	-1.35	0.54	-2.50	0.082
Gastrointestinal symptoms	0.55	0.52	1.06	0.316	Gastrointestinal symptoms	0.14	0.60	0.23	0.823
Sleep	-0.07	0.14	-0.48	0.641	Sleep	-0.14	0.28	-0.52	0.622
Observations	15				Observations	13			
R^2^/R^2^ adjusted	0.740/0.596				R^2^/R^2^ adjusted	0.618/0.346			

The relation of T0-T14 change value of the SP amplitude and T0-T14 change scores of behavioral measures in children with ASD. Significance is labeled with *p < 0.05, **p < 0.01, ***p < 0.001 (significant *p*-values are FDR-corrected) and highlighted in bold.

## 3 Results

### 3.1 Changes in main behavioral measures during intervention

Behavioral outcomes from the broader feasibility study have been reported previously (44). Because the present EEG analyses included only participants with EEG recordings meeting predefined quality criteria, the behavioral analyses were repeated in this analytical subset to confirm that the overall pattern of behavioral changes observed in the full cohort was preserved and to provide the clinical context for the electrophysiological analyses.

In order to investigate whether the administration of Bio-K+ probiotic food potentially improves clinical and behavioral characteristics of children with ASD, we fitted linear mixed-effects models with behavioral measures as dependent variables, timepoints as predictors (fixed effects) and participants as a random intercept. The models were specified *a priori* with T0 as the reference timepoint; therefore, the model coefficients directly estimated the planned comparisons of T14 vs. T0 and T22 vs. T0. The results showed that after 14 weeks of intervention (by T14), there was a significant improvement in all assessed caregiver-reported behavioral outcomes; however, no such improvement was detected at T22 (**Table 2**, **Figure 1A**).

**Figure 1:**
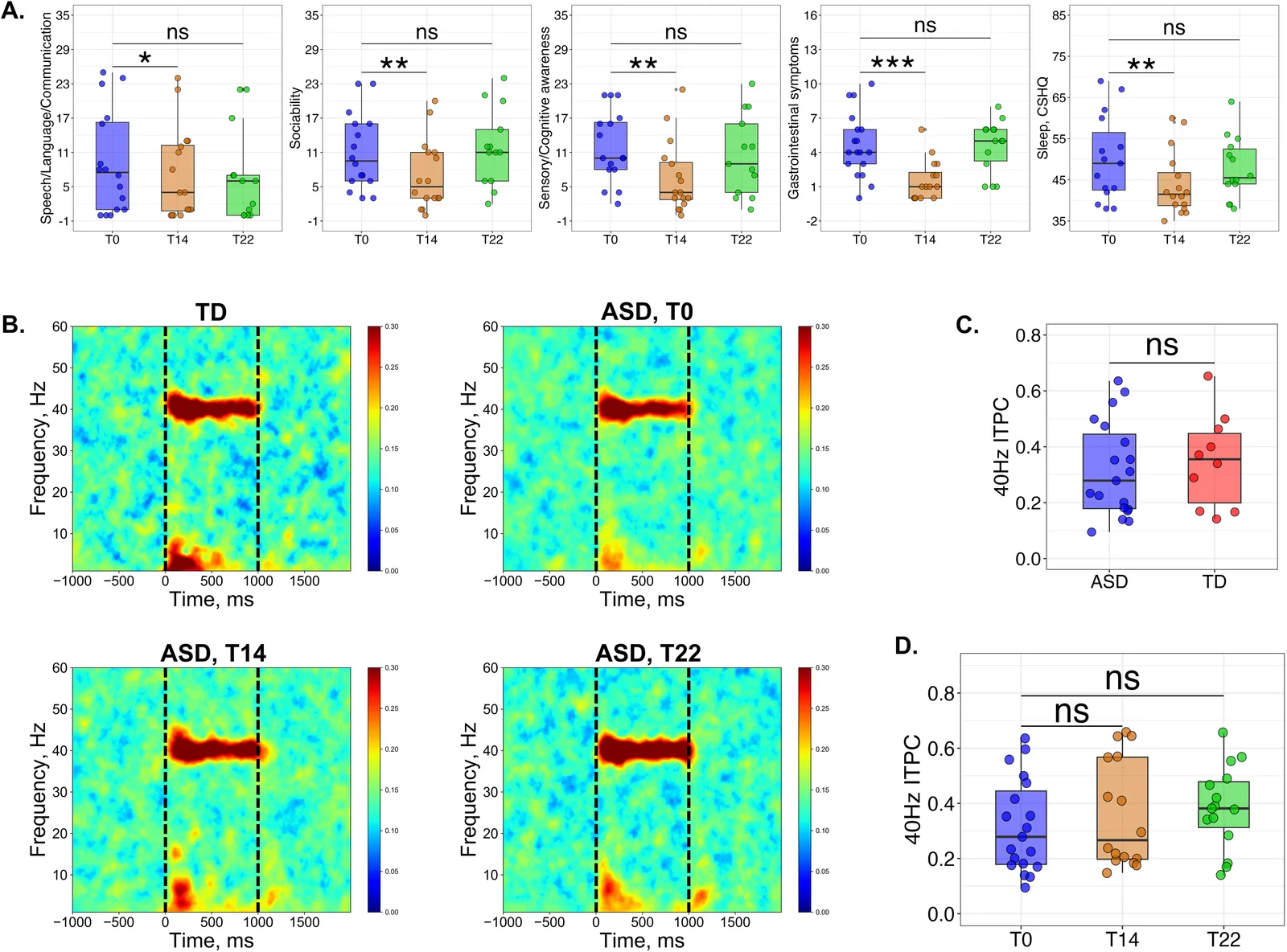
40Hz Auditory Steady-State Response (ASSR) in children with Autism Spectrum Disorder (ASD) and typically-developing (TD) controls; changes in behavioral measures during treatment in children with ASD: **(A)** Differences in behavioral measures at three timepoints in children with ASD; **(B)** Time-frequency maps for both groups of children at all timepoints (ASSR Inter-Trial Phase Coherence, ITPC), dashed lines represent stimulus start and end; **(C)** Between-group differences in 40Hz ITPC at T0; **(D)** Differences in 40Hz ITPC at three timepoints in children with ASD. Significance is labeled with *p < 0.05, **p < 0.01, ***p < 0.001, ns = non-significant (significant *p*-values are FDR-corrected). The figures are reproduced from the authors’ own preprint: Arutiunian et al. (74) license CC-BY-NC-ND 4.0, doi: 10.1101/2025.10.22.25338568.

### 3.2 40Hz ASSR: group difference, relation to clinical phenotype, and changes during intervention

To provide between-group comparison in 40Hz ITPC at T0, we used Wilcoxon rank-sum test. The results did not show a significant difference, *M*_ASD_ = 0.31 (*SD* = 0.17) vs. *M*_TD_ = 0.34 (*SD* = 0.16), *W* = 95, *p* = 0.67, 95% C.I. [–0.18, 0.10], suggesting non-altered auditory gamma response in children with ASD (**Figures 1B, C**).

To test whether 40Hz ITPC in the ASD group at T0 was associated with behavioral measures, we fitted a linear model with the neural response as dependent variable and included five predictors (behavioral assessment of speech/language/communication, sociability, sensory/cognitive awareness, GI, and sleep) as main effects. The results demonstrated no significant relationships for any measures (**Table 3**).

To explore whether the administration of Bio-K+ probiotic food influenced 40Hz ASSR in children with ASD, we utilized a liner mixed effect model consisted of 40Hz ITPC as a dependent variable, timepoint as predictor as fixed effects and participants as a random intercept. The results did not show any changes in the auditory gamma response at any timepoint when comparing to T0: T14, Est. = 0.03, SE = 0.02, *t* = 1.68, *p* = 0.103; T22, Est. = 0.02, SE = 0.02, *t* = 1.38, *p* = 0.178 (**Figure 1D**).

Finally, we tested if the changes in 40Hz ITPC were related to changes in behavioral measures in children with ASD during treatment time (from T0 to T14). The results did not reveal significant relationships between variables (**Table 3**). The negative adjusted R² suggests that the fitted model explained little of the variance in ASSR change, consistent with the exploratory nature of this analysis.

To summarize, the analysis of 40Hz ASSR – low-level auditory gamma response – showed neither a between-group (ASD vs. TD) difference, nor a relationship to clinical phenotype and changes during the administration of Bio-K+ probiotic food in children with ASD.

### 3.3 Sustained potential: group difference, relation to clinical phenotype, and changes during intervention

The amplitude of SP was significantly reduced in children with ASD in comparison to TD controls, *M*_ASD_ = –2.79 (*SD* = 6.02) vs. *M*_TD_ = –6.29 (*SD* = 3.26), *W* = 140, *p* = 0.03, 95% C.I. [0.24, 5.93], although the shape of the wave was similar to that in TD children, with the clear presence of the obligatory transient auditory components (**Figures 2A–C**).

**Figure 2:**
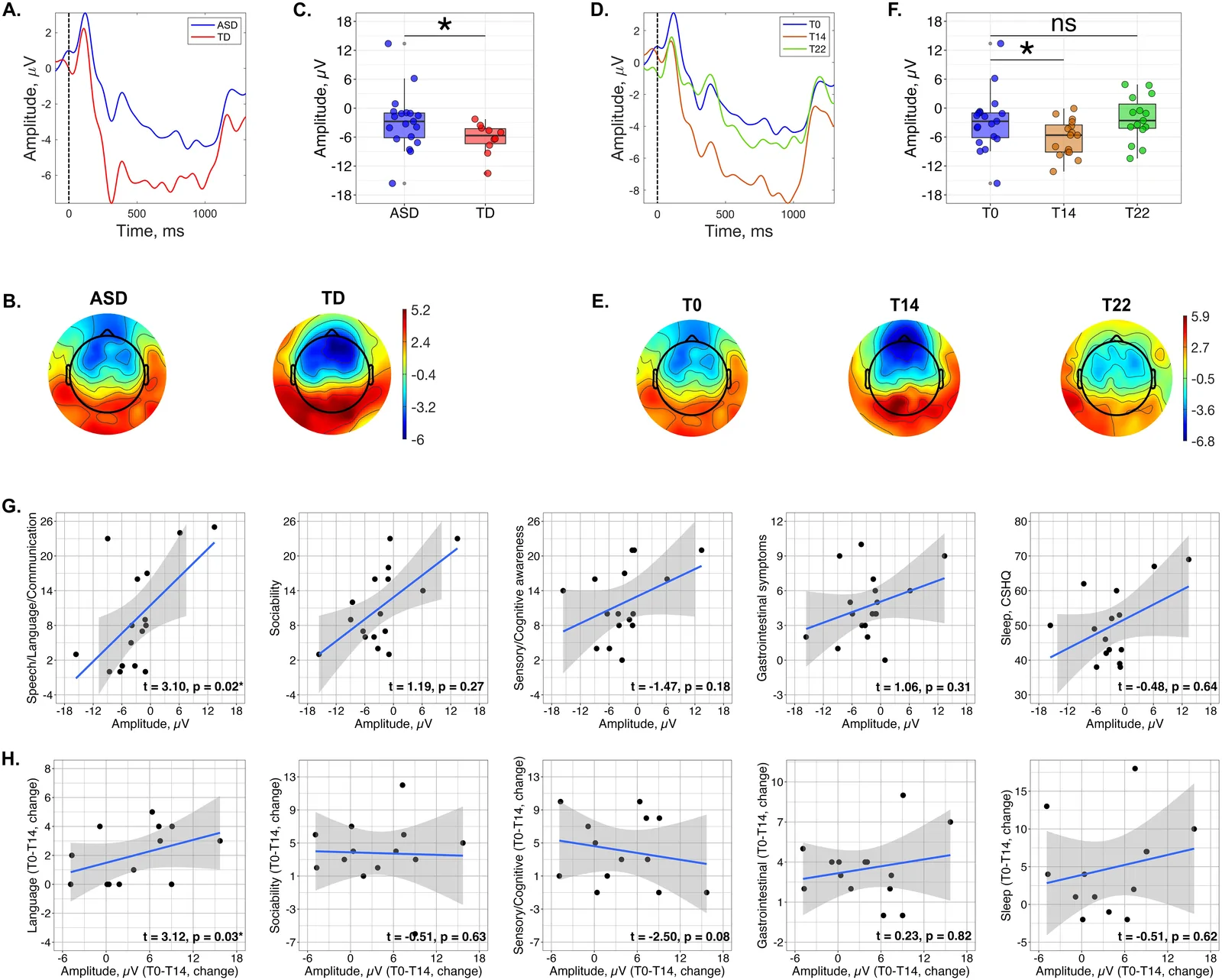
Auditory sustained potential (SP) in children with Autism Spectrum Disorder (ASD) and typically-developing (TD) controls (for SP, more negative values indicate larger response amplitudes): **(A)** Timecourses of SP in both groups of children at T0 (dashed line represents the stimulus onset); **(B)** The topographic distributions show the mean amplitude of SP in both groups of children in the 200–600 ms time window at T0; **(C)** Between-group differences in the amplitude of SP at T0; **(D)** Timecourses of SP at three timepoints in children with ASD (dashed line represents the stimulus onset); **(E)** The topographic distributions show the mean amplitude of SP at three timepoints in children with ASD in the 200–600 ms time window; **(F)** Differences in the amplitude of SP at three timepoints in children with ASD; **(G)** Relationships between SP and behavioral measures in children with ASD at T0; **(H)** Relationships between changes in SP and behavioral measures [T0-T14] in children with ASD. Significance is labeled with *p < 0.05, **p < 0.01, ***p < 0.001, ns = non-significant (significant *p*-values are FDR-corrected). The figures are reproduced from the authors’ own preprint: Arutiunian et al. (74) license CC-BY-NC-ND 4.0, doi: 10.1101/2025.10.22.25338568.

To explore whether the reduction of the SP amplitude had a clinical relevance in children with ASD, we provided brain-behavior modeling at T0 and fitted a linear model with the neural response as dependent variable and included five predictors (behavioral assessment of speech/language/communication, sociability, sensory/cognitive awareness, GI, and sleep) as main effects. The results showed a statistically significant relationship between the amplitude of SP and ATEC speech/language/communication score, Est. = 0.72, SE = 0.23, *t* = 3.10, *p* = 0.025 (**Figure 2G**, **Table 4**), indicating that the larger amplitude reduction (less negative amplitude) was associated with lower skills. Other predictors were not significant (see **Table 4**).

The next step of the analysis aimed to investigate a possible impact of the Bio-K+ probiotic product administration on the amplitude of SP in children with ASD. We used a linear mixed-effects model with SP as a dependent variable, timepoint as predictor as fixed effects and participants as a random intercept. The results demonstrated a significant change in the amplitude of the auditory response by T14 (i.e., after 14 weeks of intervention, end of the supplementation period) in comparison to T0: Est. = –3.51, SE = 1.34, *t* = –2.61, *p* = 0.027 (**Figures 2D–F**). No such change was detected for T22 when compared to T0, Est. = 0.75, SE = 1.37, *t* = 0.55, *p* = 0.586 (**Figures 2D–F**). This suggests that the amplitude of SP became significantly more negative by T14, thus, moving toward a more ‘typical’ shape (from T0 to T14, that is, during the intervention period). However, by T22 (after treatment period) it turned to a shape similar to that observed at T0.

A follow-up exploratory analysis compared the amplitudes of SP at T14 and T22 with the SP amplitude of TD children. At T14, we did not find a significant difference between the groups, *M*_ASD_ = –6.21 (*SD* = 3.73) vs. *M*_TD_ = –6.29 (*SD* = 3.26), *W* = 79, FDR-corrected *p* = 0.98, 95% C.I. [–3.34, 3.39]. However, at T22, the ASD group, similar as at T0, had a reduced amplitude of SP, *M*_ASD_ = –2.21 (*SD* = 4.68) vs. *M*_TD_ = –6.29 (*SD* = 3.26), *W* = 116, FDR-corrected *p* = 0.04, 95% C.I. [0.58, 8.04]. Therefore, indeed, after 14 weeks of intervention (by the end of the supplementation period) the amplitude of SP in children with ASD became more similar to that observed in TD controls and was no longer significantly different between the groups.

Finally, we tested whether the changes in the amplitude of SP were related to changes in behavioral measures in the ASD group during the time of Bio-K+ probiotic product administration (from T0 to T14). The output of the model is presented in **Table 4**. The results showed a significant relationship between the changes in the amplitude of SP and changes in speech/language/communication scores, such that the larger change in the SP toward more negative amplitude (and, thus, toward more ‘typical’ shape) was associated with the improvement in behavioral scores, Est. = 3.39, SE = 1.08, *t* = 3.13, *p* = 0.033 (**Figure 2H**). Other relationships were not significant.

In summary, the analysis of the SP amplitude revealed, first, its reduction in children with ASD when compared to TD controls, and this reduction was associated with lower speech/language/communication skills. Second, the amplitude of SP significantly changed during the intervention period and, by T14, was no longer significantly different from that observed in TD children. Finally, these changes in the amplitude of SP were associated with changes in ATEC speech/language/communication score toward improvement. Importantly, we demonstrated that this biomarker as well as its change was specifically related to speech/language/communication domain, but not to other behavioral measures.

## 4 Discussion

The present study aimed to investigate whether 40Hz ASSR and SP could serve as biomarkers of language impairment in children with ASD and could be utilized as objective measures of the changes in speech/language/communication skills during intervention in clinical trials. Overall, our findings provided preliminary evidence that SP may represent a promising biomarker associated with speech/language/communication domain in ASD. We observed longitudinal changes in SP amplitude over the course of the supplementation period, and these changes were related to changes in caregiver-reported speech/language/communication scores. However, given the absence of a placebo-controlled ASD group, these findings should be interpreted with caution.

Between-group comparisons (ASD vs. TD) demonstrated no difference in 40Hz steady-state gamma response at T0, which corresponds to (31–34) but not to (27–30). These inconsistencies in the previous findings can be explained by the highly heterogeneous nature of the ASD population and potential molecular mechanisms that could be altered in specific subgroups of autistic individuals (36). For example, animal studies have shown that the occupancy of *N*-methyl-D-aspartate (NMDA) receptors on PV+ interneurons reduced 40Hz auditory gamma response and, thus, alterations in this neural response may be associated with a specific subgroup within the autistic cohort but not with the whole ASD population (36, 37). In addition, methodological factors, including the relatively small sample size, developmental and clinical heterogeneity of the ASD cohort, and other study-specific factors, may also have contributed to the absence of significant ASSR findings. By contrast, we revealed a difference between groups in SP with the evidence of reduced amplitude in children with ASD. Our findings confirmed the previous studies that showed a lower amplitude of SP in children with ASD (31, 34) and Rett Syndrome (38).

Specific alterations in SP, but not 40Hz ASSR, can shed light into particular mechanisms of auditory processing that are impaired in ASD. Electrophysiological recordings in animals and MEG studies in children and adults consistently demonstrated that SP and 40Hz ASSR are generated by different cortical areas inside the Heschl’s gyrus: while both responses reflect low-level auditory processing (43), 40Hz ASSR is generated by the primary auditory cortex (‘core auditory area’) whereas neural generators of SP is in the anterolateral region of Heschl’s gyrus which represents the so-called ‘pitch processing center’ (23, 34, 39–42). Given these differences, ASSR reflects a primary neural response to specific frequency of stimulation (= 40Hz)/’pure’ tone processing based on tonotopic organization of A1, whereas SP is a more complex response that integrates pitch information across different frequency units (40) and can be more essential for speech perception and processing in comparison to ASSR. It should be noted, however, that these anatomical and functional interpretations are based on previous animal, MEG, and intracranial studies, as the present scalp EEG study did not include source localization analyses and therefore cannot directly identify the cortical generators of these responses.

Our results showed a relationship between the amplitude of SP (but not 40Hz ASSR) and speech/language/communication skills in children with ASD, such that a larger reduction of the amplitude was associated with the lower skills. A number of studies in humans using implanted electrodes and intracranial EEG with direct electrophysiological recordings in different parts of Heschl’s gyrus as well as lesion-symptom mapping studies have revealed functional differences between posteromedial (a neural generator of ASSR) vs. anterolateral (a neural generator of SP) regions of Heschl’s gyrus in relation to speech and nonspeech sound processing (58–65). For example, during naturalistic speech perception and intracranial EEG recordings across Heschl’s gyrus it has been shown that phonemic encoding increases toward anterolateral region of Heschl’s gyrus as well as this region demonstrated the highest sensitivity/preferentially responded to speech over non-speech sounds (62). It has been revealed that the posteromedial region of Heschl’s gyrus which represents the ‘core area’/A1 is involved in the processing of basic acoustic features of sounds (including speech sounds) by decomposing them into pure tones, whereas anterolateral region of Heschl’s gyrus is involved in more complex integrative (but still low-level) sound processing (63). The observed relationship between the amplitude of SP and the ATEC speech/language/communication score in children with ASD was domain specific, as no associations were found between this neural response and other behavioral measures.

Importantly, our results demonstrated the influence of the probiotic treatment on SP as well as on the changes between the amplitude of SP in relation to speech/language/communication skills in children with ASD. First, we showed that SP amplitude became significantly more negative during probiotic supplementation and, by T14, was no longer significantly different from that of TD children. However, at T22 (post-supplementation follow-up assessment stage, 8 weeks after the end of intervention), SP amplitude became less negative again, returning to baseline levels, and was again significantly different from the TD group. Second, our findings demonstrated that the changes in the amplitude of SP were associated with changes of speech/language/communication skills by the end of intervention (from T0 to T14). Specifically, a larger change in the SP toward a more negative amplitude (and, thus, toward more ‘typical’ shape) was related to improvements in behavioral scores. It is important to highlight that, although all behavioral measures (speech/language/communication; sociability; sensory/cognitive awareness; GI symptoms; sleep issues) changed during treatment and improved by T14, only the changes in speech/language/communication were related to changes of the amplitude of SP, pointing to a domain-specific relationship.

Our open-label pilot study adds to the growing body of literature investigating the potential effects of probiotic supplementation in ASD. Several individual studies have reported improvements in autistic symptomatology and co-occurring gastrointestinal and sleep problems following probiotic supplementation (66–71). Probiotic intake has been proposed to restore microbial homeostasis and improve neurobehavioral, gastrointestinal, and sleep-related outcomes through modulation of the microbiota–gut–brain axis (68). However, it is important to note that the evidence supporting these effects remains inconsistent. Recent systematic reviews and meta-analyses have highlighted substantial heterogeneity across probiotic formulations, treatment protocols, study populations, and outcome measures, with generally modest, pooled effects and overall low certainty of the available evidence (9). Therefore, the findings of the present open-label pilot study should be interpreted cautiously and viewed as preliminary until confirmed in larger, well-designed randomized placebo-controlled trials.

It is known that changes in the gut microbiome can affect neural activity through the microbiota-gut-brain axis as the neuroactive gut metabolites can modulate brain activity directly, via the systemic circulation, or via vagal and spinal afferents (72, 73). Within this context, our findings provide preliminary evidence that longitudinal changes in SP amplitude during supplementation with the Bio-K+ probiotic (*L. acidophilus* CL1285, *L. casei* LBC80R and *L. rhamnosus* CLR2) were associated with changes in caregiver-reported speech/language/communication functioning in children with ASD. However, these findings should be considered exploratory and require replication in larger, randomized, placebo-controlled studies before the clinical utility of SP as a language biomarker can be established. Furthermore, additional studies are needed to establish key biomarker properties, including test–retest reliability, developmental stability, construct validity, reproducibility across independent cohorts, and sensitivity to clinically meaningful change.

Despite its preliminary nature and open-label design, the present study has several important strengths. It is, to our knowledge, the first longitudinal study to evaluate both ASSR and SP within the same experimental paradigm in the context of an intervention study in ASD. The use of a passive EEG paradigm, which does not require active behavioral responses, together with the longitudinal design and the simultaneous assessment of electrophysiological and caregiver-reported behavioral measures, provides a valuable framework for future studies investigating objective neural outcome measures in ASD interventions.

## 5 Limitations

The study has some limitations that should be highlighted. First, the open-label design and the absence of a placebo-controlled ASD group preclude causal conclusions regarding the effects of probiotic supplementation on the observed longitudinal changes. Second, the relatively small sample size and developmental heterogeneity of the participants may limit the generalizability of the findings and statistical power. The behavioral outcomes were based on caregiver-reported measures, and direct standardized assessments of receptive and expressive language abilities were not available. In addition, formal hearing assessments were not conducted; therefore, although participants were able to complete the auditory EEG paradigm, the potential influence of individual differences in hearing status on the electrophysiological measures cannot be fully excluded. Information regarding concurrent interventions, including behavioral, educational, speech-language, or pharmacological interventions, was also not systematically collected, and their potential contribution to longitudinal changes therefore cannot be excluded. Given the exploratory nature of the analyses, the findings require replication in larger, appropriately controlled studies. More broadly, alternative explanations for the observed longitudinal changes, including regression to the mean, session-to-session variability, spontaneous fluctuations, and other methodological factors, cannot be excluded.

## 6 Conclusions

In conclusion, this open-label pilot study provides the first preliminary evidence supporting an association between SP amplitude and caregiver-reported speech/language/communication functioning in children with ASD, suggesting that SP may represent a promising candidate EEG biomarker. The amplitude of this neural response was reduced in children with ASD, and this reduction was related to speech/language/communication skills; the response became more negative during the intervention period and, by T14, was no longer significantly different from that observed in TD children. At the post-supplementation follow-up assessment, the SP amplitude became less negative again and returned toward baseline levels. Notably, larger changes toward more negative SP amplitude during the intervention period were associated with improvements in caregiver-reported speech/language/communication scores.

Therefore, our results provide preliminary evidence supporting SP as a potential EEG-based objective measure of caregiver-reported speech/language/communication functioning in children with ASD that can be used in future clinical trials, as longitudinal changes in SP amplitude over the course of the intervention period were associated with changes in behavioral scores. Moreover, because this is a passive EEG paradigm that does not require active behavioral responses or task performance, it may be particularly suitable for future studies involving non-verbal or minimally verbal individuals with profound autism, although this potential application was not directly evaluated in the present study. These promising results, although in a relatively small sample size, highlighted the importance of using this biomarker in larger, randomized, double-blind, placebo-controlled clinical trial studies.

Future studies should focus on validating SP in larger, preregistered, randomized, double-blind, placebo-controlled clinical trials using repeated electrophysiological assessments together with standardized language measures, blinded clinical outcome assessments, and formal evaluation of biomarker reliability. In addition, future studies would benefit from testing this biomarker in other clinical populations to reveal whether SP is a universal neural marker of language functioning or is specific to language impairment in children with ASD.

## Data Availability

The raw data supporting the conclusions of this article will be made available by the authors, without undue reservation.
